# Sleep Deprivation Is Associated with Bicycle Accidents and Slip and Fall Injuries in Korean Adolescents

**DOI:** 10.1371/journal.pone.0135753

**Published:** 2015-08-17

**Authors:** So Young Kim, Songyong Sim, Sung-Gyun Kim, Hyo Geun Choi

**Affiliations:** 1 Department of Otorhinolaryngology-Head & Neck Surgery and Cancer Research Institute, Seoul National University College of Medicine, Seoul, Republic of Korea; 2 Department of Statistics, Hallym University, Chuncheon, Republic of Korea; 3 Department of Internal Medicine, College of Medicine, Hallym University, Anyang, Republic of Korea; 4 Department of Otorhinolaryngology-Head & Neck Surgery, Hallym University Sacred Heart Hospital, Anyang, Republic of Korea; University of Rome, ITALY

## Abstract

**Objective:**

This study sought to evaluate associations between sleep time and bicycle accidents, falls under various circumstances, and dental injuries in adolescents.

**Methods:**

A total of 61,696 participants ranging from 12 to 18 years of age who completed the Korea Youth Risk Behavior Web-based Survey (KYRBWS) in 2013 were enrolled in this study. Bicycle riding accidents were analyzed for 17,232 bicycle-riding participants. Data were collected regarding the weekday sleep duration for the most recent 7 days, which was categorized as < 5.5 h, 5.5–6.5 h, 6.5–7.5 h, or ≥ 7.5 h per day, and the incidence of bicycle accidents, slips and falls under various circumstances, and dental injuries in the most recent 12 months. Adjusted odds ratios (aORs) were calculated among sleep groups for bicycle accidents, slips and falls, and dental injuries using simple and multiple logistic regression analyses with complex sampling.

**Results:**

Bicycle riding accidents and slips and falls in classrooms, corridors, the ground, toilets, stairs, and other unspecified situations showed positive correlations with sleep deprivation. Comparisons of groups with ≥ 7.5 h sleep, < 5.5 h, 5.5–6.5 h sleep, and 6.5–7.5 h sleep revealed increased associations with slips and falls under various circumstances. In particular, the aORs were higher in the groups with less sleep (aOR of the 5.5 h group > the 5.5–6.5 h group > the 6.5–7.5 h group). There was no significant relationship between sleep deprivation and dental injury.

**Conclusions:**

This study demonstrated that sleep deprivation among Korean adolescents was associated with bicycle accidents and falls at home and school. Thus, adequate sleep may be needed to prevent accidents and falls.

## Introduction

Many adolescents suffer from insufficient sleep. Recently, advances in computer technology and mass media have resulted in greater exposure to nighttime screen use and social networking [1]. In addition, academic burdens force adolescents to remain awake until late at night and then wake early in the morning for early school start times. It has been estimated that the average sleep time for American adolescents is approximately 7.5–8.5 h per day, which is less than 6.5 h on school days [2]. Korean adolescents are more sleep deprived at approximately 4.86–5.62 h per day, which is mainly attributed to academic demands or stress and early school start times [3].

Although there are conflicting opinions, it is generally accepted that adolescents require at least 8.5 h of uninterrupted sleep for proper functioning [4–6]. More specifically, the National Sleep Foundation has suggested that the appropriate sleep duration for adolescents is 8 to 10 h [7]. Recent studies have suggested that sleep deprivation and sleepiness in sequence increase the risks of obesity, asthma, drowsy driving and motor vehicle accidents [8–12]. In fact, some pathologic conditions have a reciprocal causal relationship with sleep. For instance, sleep deprivation may aggravate or cause mood disorders [13]. Conversely, mood disorders, particularly major depressive disorders, may lead to sleep deprivation [14].

In particular, the sleep deprivation of adolescents may provoke serious problems for physical and emotional health, academic success, and safety [13, 14]. Several recent studies have shown that sleep deprivation or sleep disturbance is associated with the risk of injury among children or adolescent athletes [15–17]. However, there have been only a few studies on the effects of sleep time on accidents and falls among adolescents. In addition, to our knowledge, no study has explored the relationship between sleep time and specific types of accidents and falls at home and school for adolescents. Therefore, we conducted a cross-sectional population-based study that primarily focused on the effects of sleep duration on bicycle accidents and falls under various circumstances among Korean adolescents.

## Materials and Methods

### Ethical considerations

The ethics committee of the Korea Centers for Disease Control and Prevention (KCDC) approved the survey. Written informed consent was obtained from each student’s parent prior to participation.

### Study Population and Data Collection

This report describes the results of a cross-sectional study that used data from the Korea Youth Risk Behavior Web-based Survey (KYRBWS). The analysis used statistical methods based on designed sampling and adjusted weight values. Data from the KYRBWS, conducted in 2013, were analyzed. All data were collected by the KCDC. Korean adolescents from 7^th^ through 12^th^ grades completed the self-administered questionnaire voluntarily and anonymously. The validity and reliability of the KYRBWS have been documented by other studies [18, 19]. The surveys evaluated data from South Korean adolescents using stratified, two-stage (schools and classes) clustered sampling based on data from the Education Ministry. Sampling was weighted by statisticians who performed post-stratification and considered non-response rates and extreme values.

Among a total of 72,047 participants, we excluded the following participants from this study: those who did not fill out the sleep time question or who had less than 2 h sleep (8,970 participants) and those who did not list their height or weight (1,381 participants). Finally, 61,696 participants (30,810 male; 30,886 female) ranging from 12 to 18 years of age were included in this study. Because 44,464 participants did not ride a bicycle to commute to school, bicycle accidents were analyzed for only 17,232 participants.

### Survey

The times of falling asleep and waking up over the most recent 7-week days were measured in hours and 10-minute increments. The duration of sleep time was calculated by subtracting the time of falling asleep from the time of waking up. To analyze the relationship between various types of injuries, the duration of sleep time per day was categorized as follows: < 5.5 h, 5.5–6.5 h, 6.5–7.5 h, and ≥ 7.5 h. To confirm the correlation between subjective sleep insufficiency and surveyed sleep duration, the degree of recovery from fatigue by sleep during the most recent 7 days was investigated and categorized as follows: 1: very sufficient, 2: sufficient, 3: not very well, 4: not sufficient, 5: very insufficient.

Days of physical activity were measured as days of exercise lasting longer than 60 minutes (i.e., sufficient to increase the heartbeat or respiration) over the most recent 7 days. Obesity was categorized into the following 4 groups according to the Centers for Disease Control and Prevention guidelines for body mass index (BMI, kg/m^2^) in children and teens (Centers for Disease Control and Prevention. About BMI for Children and Teens): obese ≥ 95^th^ percentile, overweight ≥ 85^th^ percentile and < 95^th^ percentile, healthy weight ≥ 5^th^ percentile and < 85^th^ percentile, and underweight < 5^th^ percentile. The region of residence was divided into 3 groups according to administrative district: large city, small city, and rural area. Economic level was measured as 5 levels ranging from highest to lowest.

The participants were asked about their accident history over the previous 12 months, including accidents from riding a bicycle to commute to school, slips and falls in the classroom, corridors, the ground, toilets, stairs, and other places and tooth fractures due to exercise or accident.

### Statistical Analysis

Differences in general characteristics according to each accident were calculated using independent t-tests for age, sleep time, and days of physical activity; the Chi-square test for sex; and the linear by linear association test for obesity, economic level of the house, region of residence, and recovery from fatigue.

Adjusted odds ratios (aORs) for sleep time in relation to each type of injury were calculated using simple logistic regression analysis with complex sampling (unadjusted), multiple logistic regression analysis with complex sampling adjusted for age and sex (model 1), or multiple logistic regression analysis with complex sampling adjusted for age, sex, obesity, economic level, and region of residence (model 2).

Two-tailed analyses were conducted, and *P*-values less than 0.05 were considered as statistically significant; 95% confidence intervals (CIs) were also calculated. After applying the weighted values recommended by KYRBWS, all results are presented as weighted values. The results were statistically analyzed using SPSS v. 21.0 (IBM, Armonk, NY, USA).

## Results

The average sleep time was 6.45 ± 1.39 h among the total participants. According to sleep time group, 23.8% (14,668), 24.1% (14,889), 25.1% (15,461) and 27.0% (16,678) of the participants reporting < 5.5 h, 5.5–6.5 h, 6.5–7.5 h, and ≥ 7.5 h of sleep time, respectively. The sleep time was significantly correlated with the degree of recovery from fatigue during the most recent 7 days (S1 Table).

We evaluated differences in general characteristics between the control (no accidents or slips and falls) and accident groups (Tables 1, 2 and 3).

**Table 1:** General Characteristics.

	Slip or Fall Down								
	Bicycle			Classroom			Corridor		
	No	Yes	P-value	No	Yes	P-value	No	Yes	P-value
Number (n)	14,993	2,239		60,301	1,395		58,812	2,884	
Number (%)	87	13		97.7	2.3		95.3	4.7	
Age (year)	14.81	14.5	<0.001*	14.96	14.45	<0.001*	14.97	14.46	<0.001*
Sleep Time (h) (%)			<0.001*			0.116			0.116
<5.5h (n)	3,093	406		14,315	353		14,013	655	
(%)	88.4	11.6		97.6	2.4		95.5	4.5	
5.5≤,<6.5h (n)	3,396	442		14,570	319		14,213	676	
(%)	88.5	11.5		97.9	2.1		95.5	4.5	
6.5≤,<7.5h (n)	3,808	607		15,091	370		14,699	762	
(%)	86.3	13.7		97.6	2.4		95.1	4.9	
≥7.5h (n)	4,696	784		16,325	353		15,887	791	
(%)	85.7	14.3		97.9	2.1		95.3	4.7	
Sleep time (h)	6.61	6.78	<0.001*	6.46	6.38	0.045*	6.45	6.47	0.491
Physical Exercise (d)	2.16	2.55	<0.001*	1.87	2.17	<0.001*	1.86	2.23	<0.001*
Sex (%)			<0.001†			0.264			<0.001†
Male	83.4	16.6		97.8	2.2		95.8	4.2	
Female	94.5	5.5		97.7	2.3		94.9	5.1	
Obesity (%)			<0.001‡			0.76			0.402
Underweight	88.6	11.4		98.1	1.9		95.4	4.6	
Healthy	87.7	12.3		97.7	2.3		95.3	4.7	
Overweight	84	16		90	2		95.4	46	
Obese	82.5	17.5		97.8	2.2		95.9	4.1	
Economic level (%)			0.724			0.036‡			0.727
Highest	84.6	15.4		96.9	3.1		94.4	5.6	
Middle High	87.2	12.8		97.4	2.6		95.1	4.9	
Middle	88	12		98.1	1.9		95.8	4.2	
Middle Low	85.9	16.1		97.6	2.4		95.1	4.9	
Lowest	93.9	13		97.3	2.7		94.1	5.9	
Region (%)			0.245			0.142			<0.001‡
Large City	87.2	12.8		97.6	2.4		95	5	
Small City	87	13		97.9	2.1		95.5	4.5	
Rural Area	86.3	13.7		97.7	2.3		96	4	

* Paired T-test, Significance at P < 0.05.

† Chi-square test, Significance at P < 0.05.

‡ Linear by linear association, Significance at P < 0.05.

**Table 2:** General Characteristics.

	Slip or Fall Down								
	Ground			Toilet			Stair		
	No	Yes	P-value	No	Yes	P-value	No	Yes	P-value
Number (n)	56,079	5,617		61,300	396		57,136	4,560	
Number (%)	90.9	9.1		99.4	0.6		92.6	7.4	
Age (year)	14.97	14.67	<0.001*	14.95	14.64	0.001*	14.97	14.7	<0.001*
Sleep Time (h) (%)			0.111			0.006*			<0.001*
<5.5h (n)	13,427	1,241		14,553	115		13,386	1,282	
(%)	91.5	8.5		99.2	0.8		91.3	8.7	
5.5≤,<6.5h (n)	13,477	1,412		14,790	99		13,784	1,105	
(%)	90.5	9.5		99.3	0.7		92.6	7.4	
6.5≤,<7.5h (n)	14,011	1,450		15,370	91		14,379	1,082	
(%)	90.6	9.4		99.4	0.6		93	7	
≥7.5h (n)	15,164	1,514		16,587	91		15,587	1,091	
(%)	90.9	9.1		99.5	0.5		93.5	6.5	
Sleep time (h)	6.45	6.47	0.276	6.46	6.25	0.003*	6.47	6.29	<0.001*
Physical Exercise (d)	1.82	2.4	<0.001*	1.87	2.36	<0.001*	1.86	2.1	<0.001*
Sex (%)			<0.001†			0.017†			<0.001†
Male	88.7	11.3		99.4	0.6		94.2	5.8	
Female	93.1	6.9		99.3	0.7		91	9	
Obesity (%)			0.573			0.314			0.706
Underweight	92.4	7.6		99.5	0.5		93.4	6.6	
Healthy	90.7	9.3		99.3	0.7		92.6	7.4	
Overweight	90.9	9.1		99.4	0.6		92.3	7.7	
Obese	91.6	8.4		99.7	0.3		93.5	6.5	
Economic level (%)			<0.001‡			0.984			0.438
Highest	89.6	10.4		99.7	0.9		97.9	8.1	
Middle High	89.8	10.2		99.4	0.6		92.4	7.6	
Middle	91.6	8.4		99.4	0.6		93.5	6.8	
Middle Low	91	9		99.3	0.7		91.9	8.1	
Lowest	91.5	8.5		99	1		91.3	8.7	
Region (%)			<0.001‡			0.263			0.36
Large City	90.2	9.8		99.4	0.6		92.5	7.5	
Small City	91.4	8.6		99.4	0.6		92.8	7.2	
Rural Area	91.7	8.3		99.2	0.8		92.6	7.4	

* Paired T-test, Significance at P < 0.05.

† Chi-square test, Significance at P < 0.05.

‡ Linear by linear association, Significance at P < 0.05.

**Table 3:** General Characteristics.

	Slip or Fall Down					
	Other			Dental Injury		
	No	Yes	P-value	No	Yes	P-value
Number (n)	60,663	1,033		58,782	2,914	
Number (%)	98.3	1.7		95.3	4.7	
Age (year)	14.95	14.82	0.018	14.94	15.04	0.003*
Sleep time (%)			0.026*			0.188
< 5.5 h (n)	14,394	274		13,944	724	
(%)	98.1	1.9		95.1	4.9	
5.5≤, < 6.5h (n)	14,648	241		14,181	708	
(%)	98.4	1.6		95.2	4.8	
6.5≤, <7.5h (n)	15,191	270		14,756	705	
(%)	98.3	1.7		95.4	4.6	
≥7.5h (n)	16,430	248		15,901	777	
(%)	98.5	1.5		95.3	4.7	
Sleep Time (h)	6.46	6.35	0.011*	6.46	6.42	0.192
Physical Exercise (d)	1.87	2.15	<0.001*	1.86	2.17	<0.001*
Sex (%)			0.001			<0.001†
Male	98.5	1.5		94.3	5.7	
Female	98.2	1.8		96.3	3.7	
Obesity (%)			0.372			0.356
Underweight	98.7	1.3		95.8	4.2	
Healthy	98.3	1.7		95.2	4.8	
Overweight	98.5	1.5		95.5	4.5	
Obese	98.7	1.3		94.8	5.2	
Economic level (%)			0.239			0.144
Highest	98.5	1.5		94.2	5.8	
Middle High	98.3	1.7		95.5	4.5	
Middle	98.4	1.6		95.6	4.4	
Middle Low	98.1	1.9		94.9	5.1	
Lowest	98.1	1.9		93.5	6.5	
Region (%)			0.001‡			0.303
Large City	98.4	1.6		95.3	4.7	
Small City	98.4	1.6		95.4	4.6	
Rural Area	97.7	2.3		94.9	5.1	

* Paired T-test, Significance at P < 0.05.

† Chi-square test, Significance at P < 0.05.

‡ Linear by linear association, Significance at P < 0.05.

In total, 2,239, 1,395, 2,884, 5,617, 396, 4,560, 1,033 and 2,914 participants had experienced bicycle-riding accidents; falls involving classrooms, corridors, grounds, toilets, stairs, or other facilities; and falls causing dental injury, respectively. A considerable number of participants had simultaneously experienced several types of falls or accidents. Furthermore, each type of fall or accident was significantly correlated with the others (S2 Table). Because of the large participant number, we found that mean sleep time was different between the control and accident groups. However, there were minimal differences without clinical significance (0.1–0.2 h difference). The accident group also reported more physical activity than the control group; therefore, this factor was considered in multiple logistic regression analysis.

We next analyzed the association between sleep time and bicycle accidents. Compared with the ≥ 7.5 h sleep group, bicycle accidents were negatively associated with the <5.5 h, 5.5–6.5 h, 6.5–7.5 h sleep groups in the unadjusted model (P = 0.02). However, there was a positive association in Model 1 and Model 2 analyses, which means that, compared with the ≥ 7.5 h sleep group, the rates of bicycle riding accidents were increased in the groups with lower sleep durations (Each P = 0.01) (Table 4).

**Table 4:** Odd ratios of less sleep time (h) for each injury by multiple logistic regression analysis with complex sampling.

Types of Injury	Unadjusted		Model 1†		Model 2‡	
Sleep Time (h)	AOR (95% CI)	P Value	AOR (95% CI)	P Value	AOR (95% CI)	P Value
**Bicycle**		0.02*		0.01*		0.01*
< 5.5 h	0.87(0.76–0.99)		1.31(1.12–1.53)		1.31(1.12–1.53)	
5.5≤, < 6.5 h	0.84(0.73–0.96)		1.14(0.99–1.31)		1.1(0.99–1.32)	
6.5≤, <7.5 h	0.99(0.87–1.12)		1.18(1.03–1.34)		1.17(1.03–1.33)	
≥7.5 h	1		1		1	
**Slip or Fall**						
**Classroom**		0.28		<0.001*		< 0.001*
< 5.5 h	1.15(0.99–1.34)		2.07(1.73–2.47)		2.02(1.69–2.42)	
5.5≤, < 6.5 h	1.05(0.90–1.23)		1.63(1.37–1.93)		1.61(1.36–1.90)	
6.5≤, <7.5 h	0.98(0.9–1.28)		1.36(1.17–1.59)		1.35(1.16–1.58)	
≥7.5 h	1		1		1	
**Corridor**		0.07		< 0.001*		<0.001*
< 5.5 h	0.89(0.80–0.99)		1.44(1.27–1.64)		1.42(1.25–1.60)	
5.5≤, < 6.5 h	0.91(0.82–1.02)		1.31(1.17–1.47)		1.29(1.15–1.45)	
6.5≤, <7.5 h	1.01(0.91–1.11)		1.19(1.07–1.32)		1.18(1.07–1.31)	
≥7.5 h	1		1		1	
**Ground**		0.03*		< 0.001*		<0.001*
< 5.5 h	0.94(0.86–1.02)		1.51(1.38–1.66)		1.50(1.37–1.65)	
5.5≤, < 6.5 h	1.06(0.97–1.15)		1.52(1.39–1.66)		1.51(1.39–1.65)	
6.5≤, <7.5 h	1.03(0.96–1.11)		1.27(1.17–1.37)		1.26(1.17–1.36)	
≥7.5 h	1		1		1	
**Toilet**		0.26		< 0.001*		<0.001*
< 5.5 h	1.30(0.99–1.72)		2.04(1.47–2.84)		2.02(1.46–2.81)	
5.5≤, < 6.5 h	1.09(0.81–1.47)		1.52(1.11–2.10)		1.52(1.10–2.09)	
6.5≤, <7.5 h	1.15(0.83–1.58)		1.34(0.96–1.87)		1.33(0.96–1.86)	
≥7.5 h	1		1		1	
**Stair**		< 0.001*		< 0.001*		<0.001*
< 5.5 h	1.32(1.22–1.44)		1.80(1.63–1.99)		1.79(1.62–1.98)	
5.5≤, < 6.5 h	1.15(1.05–1.25)		1.44(1.31–1.59)		1.44(1.30–1.58)	
6.5≤, <7.5 h	1.06(0.97–1.16)		1.17(1.06–1.28)		1.16(1.06–1.28)	
≥7.5 h	1		1		1	
**Others**		0.04*		< 0.001*		<0.001*
< 5.5 h	1.31(1.09–1.57)		1.55(1.26–1.90)		1.58(1.29–1.93)	
5.5≤, < 6.5 h	1.15(0.96–1.38)		1.31(1.08–1.59)		1.33(1.10–1.61)	
6.5≤, <7.5 h	1.21(1.01–1.46)		1.28(1.07–1.54)		1.28(1.07–1.54)	
≥7.5 h	1		1		1	
**Dental Injury**		0.47		0.52		0.49
< 5.5 h	0.99(0.90–1.11)		1.05(0.92–1.19)		1.05(0.92–1.19)	
5.5≤, < 6.5 h	0.98(0.88–1.09)		1.01(0.90–1.14)		1.01(0.90–1.14)	
6.5≤, <7.5 h	0.93(0.83–1.03)		0.96(0.86–1.07)		0.95(0.86–1.07)	
≥7.5 h	1		1		1	

* Significance at P < 0.05

† adjusted for age and sex

‡ adjusted for age, sex, physical exercise day, obesity, economic level, and region of residence.

In the unadjusted model, slips and falls under various circumstances were not definitively associated with specific sleep groups. However, in the Model 1 and Model 2 analyses, compared with the ≥ 7.5 h sleep group, the < 5.5 h, 5.5–6.5 h, and 6.5–7.5 h sleep groups showed positive associations with slip and fall accidents in all circumstances (P for each < 0.001). Moreover, we found a dose-dependent relationship between accidents and sleep time; participants with less sleep showed a higher aOR for slips and falls (aOR of 5.5 h group > 5.5–6.5 h group > 6.5–7.5 h group) (Table 4).

Dental injury was not associated with sleep time in unadjusted, Model 1, or Model 2 analysis (Table 4).

## Discussion

This study evaluated associations between bicycle riding accidents, slips and falls under various circumstances and dental injury with sleep time. Less sleep time was related to an increase in bicycle riding accidents and falls under various circumstances but was not related to dental injury. There were also significant correlations between the different types of falls (S2 Table). Our results did not vary across any of the covariates adjusted, including age, sex, days of physical exercise, obesity, economic level, and region of residence. Our study is the first to report the relationship between reduced sleep time and bicycle accidents and falls under various circumstances among adolescents.

Accidents and falls occur following inappropriate reactions to barriers or an impaired balance system. Reactions to physical obstacles to avoid accidents and falls requires advanced cognitive and self-regulatory skills associated with keen attention, adequate reaction time, precise decision making and appropriately regulated impulsivity [20, 21]; however, each of these skills can be hampered by sleep deprivation [22]. Chronically or acutely sleep-deprived individuals demonstrate increased impulsivity, slower reaction times, decreased attention, and impaired decision making [23–25].

Conflicting results have been reported for the effects of sleep deprivation on accidents and falls. One previous study demonstrated an approximately twofold increased risk for accidents among drivers with an average of 6 h or less of sleep per night in the last 3 months [26]. However, another study suggested that less than 6 h of sleep in the preceding 24 h was not associated with the risk of fall-related injuries [27], which suggests that the immediate effects of acute sleep deprivation may not be sufficient to explain the increased risk of accidents for adolescents. Adverse consequences of chronic sleep deprivation, such as psychological distress, can also have an influence on accidents for adolescents. In our survey, the typical sleep time during weekdays was collected for participants; therefore, our data represent the chronic, rather than acute, effects of sleep deprivation, and the results are in accordance with previous studies.

Several lines of evidence imply that there may be some relationship between sleep deprivation and obesity. For instance, it has been suggested that self-reported sleep time is associated with insulin resistance, which results in obesity [28, 29]. However, our data were adjusted for obesity, and we found no relationship between obesity and accidents and falls. In other words, sleep deprivation was an independent factor related to accidents and falls irrespective of obesity. In line with this finding, a previous study demonstrated a significantly increased reaction time in young versus elderly individuals [30]. The researchers suggested that young subjects are more vulnerable to sleep deprivation given their better baseline performance and their expression of a higher degree of fatigue for an identical sleep deprivation situation, compared with older subjects.

Sleep deprivation may be a sign or symptom of other factors associated with accidents and falls for adolescents. For example, it is known that sleep deficiency is associated with mental health issues, such as anxiety and stress [31]. Moreover, sleep-deprived subjects may suffer from insomnia, or chronically sleep-deprived subjects may suffer from poor sleep hygiene due to underlying psychological illnesses. One previous study reported that subjects who reported insufficient sleep also had sleep complaints, difficulty falling asleep, repeated awakening and/or premature awakening [26]. Therefore, future studies should investigate adolescents with sleep deprivation regarding the presence of hidden pathologies such as psychological illnesses, which may increase vulnerability to accidents and falls.

In our study, there was no significant association between sleep deprivation and dental injury. Tooth fracture can occur as a result of external forces from other people or stimuli, and accidents and falls are not always accompanied by dental injury. Permanent tooth injuries in adolescents are mainly accounted for by falls at home and school [32]. However, accidents during sports, violence and traffic accidents are also common causes of dental injury. In addition, children and adolescents are more susceptible to dental injuries than adults are [33]. The intrinsic weakness of dental structures and perioral anatomies, such as an overbite or inadequate lip coverage, could also influence dental injury [34]. Moreover, many external forces placed on the teeth may not be sufficiently strong enough to induce dental fractures. Therefore, dental injury may not directly reflect deprived sleep-related changes in the incidence of falls or accidents.

There were some limitations to the current study. First, this study relied on a self-reported questionnaire to document sleep time and covariates. Previous studies comparing self-reported sleep time with objective measurements obtained by actigraphy demonstrated that self-reported data often overestimate actual sleep time, implying that the problem of sleep deprivation for adolescents may actually be greater [35]. However, there is also the possibility of underestimation due to recall bias in this retrospective survey. Moreover, although the subjects had identical length of sleep time, it was possible that some subjects suffered from insomniac complaint or nocturnal awakening. These sleep disturbances might influence the quality of sleep and could affect the falls. However, there were significant correlations between self-reported sleep time and subjective sleep deprivation in the present study (S1 Table).

We surveyed weekday sleep time over the most recent 7 days. Therefore, we did not account for overcoming the weekday sleep deprivation through weekend or daytime napping. It is known that napping during the daytime or weekend may compensate for sleep deprivation through an improvement in cognitive function [36]. However, we examined usual sleep time and not acutely sleep-deprived weekdays, which minimized perturbations of sleep time caused by an acutely deprived or overwhelmed irregular sleep pattern.

Our study included sleep time groups of < 5.5 h, 5.5–6.5 h, and 6.5–7.5 h. In Korea, the mean sleep time of adolescents is 6.45 h; in our study, 23.8% of the participants slept less than 5.5 h, and 24.1% of the participants slept 5.5–6.5 h. Most of the participants slept less than 8 h, which is less than the sleep duration generally accepted as an adequate sleep time for adolescents [4–6]. Therefore, we could not evaluate the effects of excessive sleep (greater than 9 h per day) in this study.

One of the strengths of this study was the use of a representative population of Korean adolescents, which minimized sampling bias. Moreover, we excluded data with a lack of information even if just one survey item was missing. Finally, we investigated and classified specific situational accidents and falls to analyze relationships with sleep time. Because there was heterogeneity in the causes and associated factors for each accident and fall, this specified approach was superior to excluding various confounding factors.

## Conclusions

Korean adolescents suffer from sleep deprivation, with a mean sleep time of 6.45 ± 1.39 h during weekdays. In this sleep-deprived population, there were negative correlations between sleep time and bicycle riding accidents and slips and falls in classrooms, corridors, the ground, toilets, stairs, and other unclassified situations. Therefore, the identification and resolution of sleep deprivation may be potentially helpful for preventing accidents and falls among adolescents.

## Supporting Information

S1 Table Degree of recovery from fatigue according to the sleep time. (DOCX)

S2 Table Correlations between different types of fall down injuries. (DOCX)
