# Supplementary material for: Sleep Deprivation Is Associated with Bicycle Accidents and Slip and Fall Injuries in Korean Adolescents
Source: PLoS One. 2015 Aug 17;10(8):e0135753. doi: 10.1371/journal.pone.0135753 (PMC4539229; doi:10.1371/journal.pone.0135753)
Supplement: S1 Table — (DOCX) [file pone.0135753.s001.docx]

**S1 Table** Degree of recovery from fatigue according to the sleep time

| Sleep time (h) | Degree of recovery from fatigue for recent 7 days | | | | | Total |
| --- | --- | --- | --- | --- | --- | --- |
|  | 1 | 2 | 3 | 4 | 5 |  |
| < 5.5 h (n) | 299 | 993 | 3,392 | 5,942 | 4,042 | 14,668 |
| (%) | 2.0 | 6.8 | 23.1 | 40.5 | 27.6 | 100.0 |
| 5.5≤, < 6.5h (n) | 532 | 1,947 | 4,953 | 5,392 | 2,065 | 14,889 |
| (%) | 3.6 | 13.1 | 33.3 | 36.2 | 13.9 | 100.0 |
| 6.5≤, <7.5h (n) | 907 | 3,135 | 5,889 | 4,234 | 1,296 | 15,461 |
| (%) | 5.9 | 20.3 | 38.1 | 27.4 | 8.4 | 100.0 |
| ≥7.5h (n) | 2,394 | 5,290 | 5,501 | 2,743 | 750 | 16,678 |
| (%) | 14.4 | 31.7 | 33.0 | 16.4 | 4.5 | 100.0 |
| Total (n) | 4,132 | 11,365 | 19,735 | 18,311 | 8,153 | 61,696 |
| (%) | 6.7 | 18.4 | 32.0 | 29.7 | 13.2 | 100.0 |

(P < 0.001 by linear by linear analysis)

(1: very sufficient; 2: sufficient; 3: not very well; 4: not sufficient; 5: very insufficient)
