# Supplementary material for: Sleep Deprivation Is Associated with Bicycle Accidents and Slip and Fall Injuries in Korean Adolescents
Source: PLoS One. 2015 Aug 17;10(8):e0135753. doi: 10.1371/journal.pone.0135753 (PMC4539229; doi:10.1371/journal.pone.0135753)
Supplement: S2 Table — (DOCX) [file pone.0135753.s002.docx]

**S2 Table** Correlations between different types of fall down injuries.

|  |  | Classroom | Corridor | Ground | Toilet | Stair | Others | Dental Injury |
| --- | --- | --- | --- | --- | --- | --- | --- | --- |
| Classroom | r† | 1 | 0.245 | 0.161 | 0.117 | 0.155 | 0.097 | 0.023 |
|  | P-value | N/A | <0.001* | <0.001* | <0.001* | <0.001* | <0.001* | <0.001* |
| Corridor | r† | 0.245 | 1 | 0.181 | 0.142 | 0.232 | 0.079 | 0.025 |
|  | P-value | <0.001* | N/A | <0.001* | <0.001* | <0.001* | <0.001* | <0.001* |
| Ground | r† | 0.161 | 0.181 | 1 | 0.076 | 0.159 | 0.064 | 0.018 |
|  | P-value | <0.001* | <0.001* | N/A | <0.001* | <0.001* | <0.001* | <0.001* |
| Toilet | r† | 0.117 | 0.142 | 0.076 | 1 | 0.135 | 0.080 | 0.017 |
|  | P-value | <0.001* | <0.001* | <0.001* | N/A | <0.001* | <0.001* | <0.001* |
| Stair | r† | 0.155 | 0.232 | 0.159 | 0.135 | 1 | 0.064 | 0.014 |
|  | P-value | <0.001* | <0.001* | <0.001* | <0.001* | N/A | <0.001* | <0.001* |
| Others | r† | 0.097 | 0.079 | 0.064 | 0.080 | 0.064 | 1 | 0.006 |
|  | P-value | <0.001* | <0.001* | <0.001* | <0.001* | <0.001* | N/A | .131 |
| Dental Injury | r† | 0.023 | 0.025 | 0.018 | 0.017 | 0.014 | 0.006 | 1 |
|  | P-value | <0.001* | <0.001* | <0.001* | <0.001* | <0.001* | .131 | N/A |

*P ≤ 0.05, † Pearson’s correlation coefficient
